# A New Postoperative Stability Score to Predict Loss of Reduction in Intertrochanteric Fractures in Elderly Patients

**DOI:** 10.3390/life14070858

**Published:** 2024-07-09

**Authors:** Shih-Heng Sun, Chun-Yu Chen, Kai-Cheng Lin

**Affiliations:** 1 Department of Rehabilitation, Taoyuan General Hospital, Ministry of Health and Welfare, Taoyuan 330, Taiwan; willfreund303024@gmail.com; 2 School of Medicine, National Yang Ming Chiao Tung University, Taipei 112, Taiwan; 3 Department of Orthopedics, Kaohsiung Veterans General Hospital, Kaohsiung 81341, Taiwan; iergy2000@gmail.com

**Keywords:** hip fracture, geriatric, intramedullary nailing, stability score

## Abstract

The study aimed to validate a newly developed postoperative stability score for evaluating clinical follow-up in elderly patients with low-energy hip fractures. From 1 January 2020 to 31 December 2021, we enrolled patients aged over 65 who underwent cephalomedullary nail fixation using proximal femoral nail antirotation II (PFNAII) and had at least 6 months of follow-up; excluding multiple fractures, pathological fractures, and periprosthetic fractures. We collected general patient data. Parameters such as TAD, Parker’s ratio (AP and lateral), and the new postoperative stability score were recorded. A loss of reduction was defined using the decline in the Chang reduction quality criteria (CRQC) score within one month. Among the 108 enrolled patients, 23 (21.3%) experienced a loss of reduction, with a mean age of 82.1 years and a mean follow-up time of 7.4 months. Univariate analysis showed no significant association between loss of reduction and general data. However, the new postoperative stability score correlated significantly with loss of reduction (mean scores: 6.68 vs. 4.83, *p* = 0.045). Multivariate analysis confirmed this association (odds ratio: 0.076, 95% confidence interval: 0.022–0.263, *p* < 0.05). The newly developed postoperative stability score, incorporating surgical technique assessment, improves prediction accuracy for loss of reduction in elderly intertrochanteric fracture (ITF) patients.

## 1. Introduction

Hip fracture is an important medical issue, affecting at least 1.66 million people worldwide, and this is expected to increase to 4.5–6.3 million by 2050 according to the International Osteoporosis Foundation [1,2]. Mortality rates associated with hip fractures are notably higher among elderly individuals [3,4]. Numerous studies have identified the factors influencing clinical outcomes after hip fracture, including preoperative functional capacity, overall health status, and surgical technique [5,6,7,8,9]. These fractures can be categorized as intracapsular (femoral neck fractures) or extracapsular (intertrochanteric and subtrochanteric fractures). ITFs account for 42% of all hip fractures and represent 44% of the associated costs [10].

Surgery remains the primary treatment for hip fractures [11]. The choice of surgical method depends on the fracture type. Stable fractures (AO/OTA 31-A1) benefit from sliding or dynamic hip screw (DHS) systems providing rigid fixation [12]. In contrast, intramedullary nails have become the preferred implant for unstable fractures (AO/OTA 31-A2, A3), surpassing DHSs [13,14,15]. Notably, the DHS is associated with a higher risk of postoperative mechanical failure compared to intramedullary nails [16,17,18]. 

The proximal femur nail anti-rotation II (PFNA II DePuy Synthes, Oberdorf, Switzerland) is the preferred choice for treating ITF at our hospital. Numerous meta-analyses have consistently shown that PFNA II offers advantages over the DHS in terms of reduced fixation failure, decreased blood loss, and shorter hospital stays [19,20]. However, it is important to note that some studies have reported mechanical failures in ITFs treated with PFNA II [21,22]. 

In addition to the well-known risk factors proposed by the article—such as a TAD greater than 25 mm [23], a varus neck-shaft angle [24], and blade position not being inferior on anteroposterior radiographs and not centrally placed on lateral radiographs [25]—there may be other factors contributing to loss of reduction. While we recognize the critical importance of reduction quality, several additional factors influence the likelihood of mechanical failure post-surgery. These factors include implant seating position, complete occupancy of the medullary canal, and the use of a lateral wall side plate to ensure integral stability. Although many of these factors remain speculative, our study aims to validate a newly developed postoperative stability score [26] for evaluating clinical outcomes in our patient series. 

## 2. Material and Methods

### 2.1. Patients

This retrospective study included 120 patients with ITF who underwent surgery at Kaohsiung Veterans General Hospital in Kaohsiung, Taiwan. The inclusion criteria comprised patients who underwent PFNAII (DePuy Synthes, Oberdorf, Switzerland) for hip fracture and had a follow-up period of more than 6 months. Exclusion criteria included patients under 65 years of age, those with multiple fractures, and patients with pathological fractures.

### 2.2. Data Collection and Assessments

**Figure 1:**
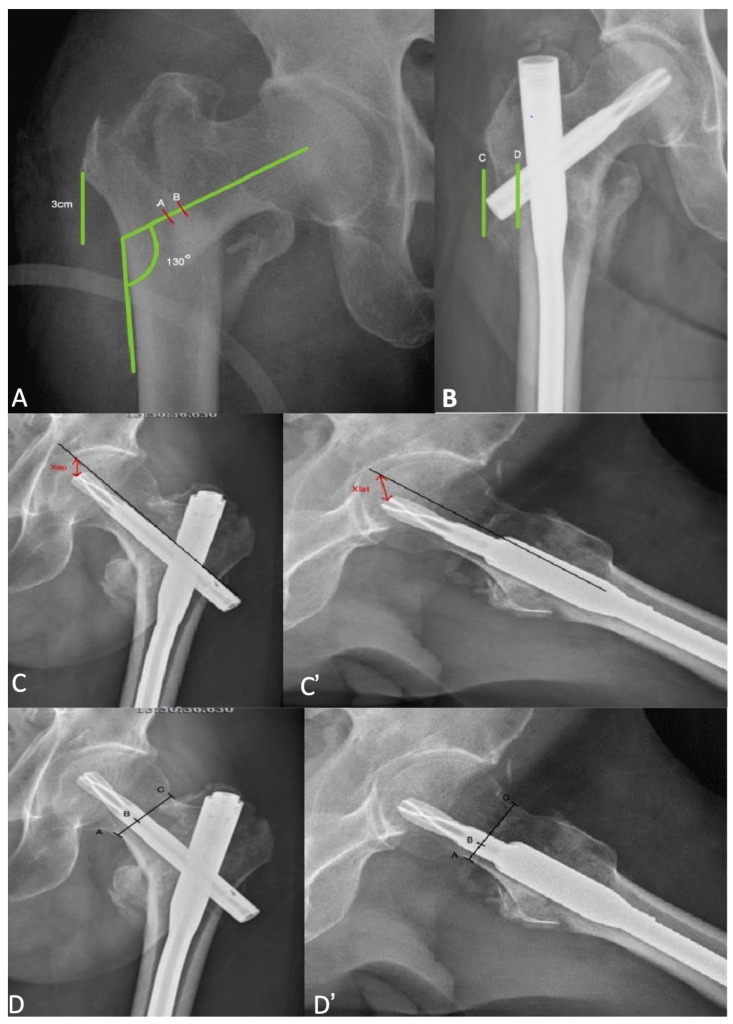
(**A**) Lateral wall fracture gap length defined as the distance in millimeters from a reference point 3 cm below the innominate tubercle of the greater trochanter, angled at 130° upward to the fracture line (distance between A and B). (**B**) Distance between the posterior tip of the blade and the lateral cortex (distances C and D). (**C**,**C’**) TAD was defined as measurement of tip apex distance and is the sum of the distance from the apex of the femoral head to the top of the lag screw on anteroposterior and lateral radiographs (Xap + Xlat). (**D**) Parker’s ratio in AP% was AB/AC. (**D’**) Parker’s ratio in lateral% was AB/AC.

The data collected included patient age, gender, BMI, ASA classification, fracture side, and fracture classification according to AO/OTA classification. Additionally, we recorded nail length, blood transfusion during the operation, time of surgery, hospital stay after surgery, time to mobilization, time to bone union, lateral wall fracture gap length, TAD, Parker’s ratio in both anteroposterior (AP) and lateral views, the distance between posterior tip of blade to lateral cortex, and the newly developed postoperative stability score (Figure 1). TAD was a measurement of tip apex distance and is the sum of the distance from the apex of the femoral head to the top of the lag screw on anteroposterior and lateral radiographs. Parker’s ratio represents the percentage of the screw from the inferior border of the femoral neck. Immediate postoperative X-rays and X-rays taken one month later were evaluated by two orthopedic surgeons. Without knowledge of the patient’s outcome, we assessed the quality of reduction using the Chang reduction quality criteria (CRQC) [27]. The CRQC considers positive and negative medial cortical support for fracture reduction and divides cases into three groups based on their scores. We defined a loss of reduction based on the decline in CRQC scores within one month. Our rationale for this definition is that displacement occurring within the first month may be related to the surgical procedure. 

### 2.3. Statistical Analysis

We conducted univariate analysis using a two-tailed independent-sample *t*-test for numerical factors and a chi-square test for categorical factors. For multivariate analysis, we employed a logistic regression model to estimate the odds ratio (OR) for loss of reduction in ITF fixation with PFNAII. All statistical analyses were carried out using IBM SPSS v.22.0 (SPSS IBM, New York, NY, USA).

## 3. Results

**Figure 2:**
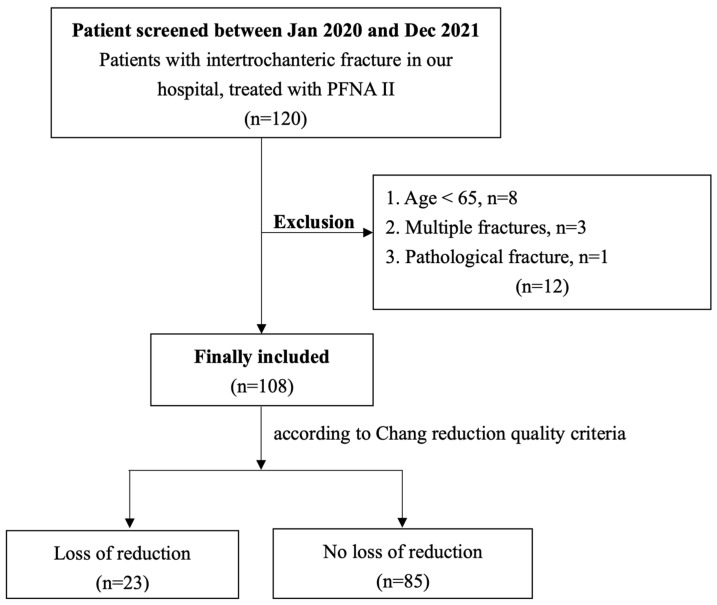
Flow chart describing patient groups and patient selection. PENA II, proximal femoral nail antirotation.

Of the 120 patients recruited, eight were younger than 65 years old, three had multiple fractures, and one had a pathological fracture. A total of twelve patients were excluded from the study. In total, 108 patients were eligible for analysis (Figure 2). The mean age of the patients was 82.1 years (range 66–97), with 76 (70.3%) being female. The median follow-up duration was 14.6 months (range 6–17). The AO/OTA fracture types included 31-A1 (21, 19.4%), A2 (61, 56.4%), and A3 (26, 24%).

Patient demographics, medical comorbidities, and radiologic parameters are summarized in Table 1. Among the patients, 23 (21.2%) presented with loss of reduction (Table 2). Univariate analysis revealed that the newly developed postoperative stability score was significantly associated with loss of reduction, with mean scores of 6.68 and 4.83, respectively (*p* = 0.045). This association remained significant in multivariate analysis, with odds ratios of 0.076 (95% confidence interval, 0.022–0.263; *p* < 0.05) (Table 3). 

## 4. Discussion

The most important findings of the study revealed no statistically significant association between loss of reduction and general data based on univariate analysis. However, the newly developed postoperative stability scores exhibited a significant correlation with loss of reduction, with mean scores of 6.68 and 4.83, respectively (*p* = 0.045). This significance persisted in multivariate analysis, with odds ratios of 0.076 (95% confidence interval, 0.022–0.263; *p* < 0.05). 

Various evaluation methods exist for assessing postoperative reduction quality, including TAD, Parker’s ratio, and the Baumgaertner reduction quality criteria (BRQC). The BRQC, developed by Baumgaertner et al. [28], places emphasis on anatomical reduction, specifically valgus neck-shaft angle and less than 4 mm of displacement. In 2015, the CRQC introduced the concept of positive medial cortical support [27], which stands in contrast to the nonanatomic positive cortex buttress reduction proposed by Gotfried [29,30]. According to Chang, positive medial cortical support indicates that the distal femoral shaft fragment is positioned laterally to the lower medial edge of the proximal fracture fragment. The CRQC primarily applies to assessing fracture reduction quality in pertrochanteric fractures treated with cephalomedullary nails such as the PFNA and Gamma nails. A retrospective study demonstrated that CRQC is reliable for predicting mechanical complications and outperforms BRQC in reliability [31]. However, there is currently no new postoperative stability score that incorporates TAD, lateral wall integrity, and other evaluation methods. Therefore, we propose using the new postoperative stability score to assist in evaluating the likelihood of post-surgery reduction loss. Notably, the new score considers implant position, including TAD and Parker’s ratio, as well as nail thickness and the integrity of the lateral femoral wall [26].

In patients with hip fractures, loss of reduction can lead to mechanical failure. To address this, Baumgaertner et al. [28] developed a quantitative measure for outcome prediction. They introduced the concept of TAD, which represents the distance between the screw tip and the femoral head apex. In 1995, they proposed TAD as a predictor of cut-out failure. Recommendations include maintaining TAD values greater than 25 mm, with the lag screw positioned centrally or inferiorly on the AP view and centrally on the lateral view. Notably, this study was based on the DHS, which differs in design from the PFNA. A systematic review encompassing various hip fracture implants confirmed the significance of TAD in cut-out failure [23]. The risk of cut-out failure was closely associated with TAD values exceeding 25 mm, consistent with Baumgaertner et al.’s findings. However, Amir Herman et al. [32] offered a different perspective. They argued that the TAD scale solely considers length measurement and neglects direction. Their study identified the “safe zone” for lag screw placement—defined as the lower 25–50% of the head–neck interface line—as the critical factor in preventing mechanical failure. Our study also revealed that TAD alone cannot reliably predict loss of reduction.

Parker’s ratio, first described by M. J. Parker in 1992 [33], guides the optimal placement of screws in femoral neck fractures. According to Parker, the screw should be positioned centrally or inferiorly on the AP view and centrally on the lateral view. Consensus suggests that the lag screw should be centrally placed in the lateral plane and either centrally or inferiorly in the AP plane [33,34,35]. Biomechanical studies have demonstrated that placing the lag screw slightly inferiorly on the AP radiograph, just above the calcar, and centrally in the femoral neck on the lateral radiograph can maximize biomechanical stiffness [25]. Through the Parker’s ratio, the location of the screw could be well explained. Numerous studies found a higher Parker’s ratio in AP radiograph was significantly associated with increased rate of cut out [33,36]. However, most studies have not found a significant association between Parker’s ratio in the lateral radiograph and cut-out rates [36]. Our study aligns with this finding, emphasizing that Parker’s ratio alone is not the sole risk factor for predicting loss of function. 

Lateral wall thickness plays a crucial role in predicting postoperative loss of reduction. Previous studies have highlighted that lateral wall fractures are most common in AO/OTA 31-A2.2 and 31-A2.3 fractures [37]. When a lateral wall fracture occurs, the screw and proximal part of the nail can slide laterally without any restraint. This lateral movement, combined with axial stress, can lead to screw cutout or loosening. CE Hsu et al. [38] investigated the reliability of lateral wall thickness as a predictor of lateral wall fracture using the DHS system. They found that a lateral wall thickness greater than 20.5 mm reliably predicts postoperative lateral wall fractures and helps prevent secondary lateral wall fractures. Due to implant design differences between DHS and PFNA, it is notable that the optimal lateral wall thickness may vary depending on the implant design. For example, Liqin Zheng et al. [39] valuated the influence of lateral wall thickness on implant failure using the PFNA system. They recommended a wall thickness of more than 21.4 mm to prevent secondary lateral wall fractures.

The treatment of ITF fractures involves several technical considerations. Fracture reduction is the crucial initial step and outweighs other factors [40]. Subsequent fracture stability relies heavily on the attachment of the anterior and medial cortex. Studies have emphasized that achieving nonanatomic positive medial cortical support during reduction can prevent relative sliding and create a more favorable environment for bone union [27,41]. Additionally, the position of the lag screw, as measured by the TAD and Parker’s ratio, plays a critical role. Furthermore, nail thickness is essential; ensuring that the canal is adequately filled can prevent sliding and provide buttress support. Lateral wall integrity is crucial, and a certain thickness is necessary to prevent secondary lateral wall fractures. Ultimately, predicting mechanical failure after surgery remains challenging due to the multifactorial nature of risk factors. All potential risks should be carefully considered. Thus, in our retrospective evaluation, we utilized a newly developed postoperative stability score [26] to assess cases. We found a significant association between this score and loss of reduction, with mean scores of 6.68 and 4.83, respectively (*p* = 0.045). Multivariate analysis further confirmed this significance, revealing odds ratios of 0.076 (95% confidence interval: 0.022–0.263; *p* < 0.05). The advantage of combining these factors lies in comprehensive evaluation. Notably, our results (as shown in Table 3) indicate that the tip–apex distance (TAD) and Parker’s ratio are not statistically significant predictors. Overall, the newly developed postoperative stability score provides valuable insights into the risk of loss of reduction.

This study has several limitations. First, due to its retrospective design, some patients were lost to follow-up during the study. Second, we employed the CRQC to assess loss of reduction. It is important to note that CRQC sets a high standard; even in cases without screw cutout or cut-in, any displacement is considered as loss of reduction. Third, increasing the number of patients could enhance the statistical power of the study.

## 5. Conclusions

The newly developed postoperative stability score, incorporating surgical technique assessment, improves prediction accuracy for loss of reduction in elderly ITF patients.

## Figures and Tables

**Table 1:** Patients’ demographics data and radiographic results.

Variable	Patients
N	108
Age (years), mean (SD)	82.1 (7.64)
Sex, n (%) Male Female	32 (29.7) 76 (70.3)
BMI, mean (SD)	23 (4.3)
ASA classification, n (%) I II III IV V VI	1 (0.92) 43 (39.8) 60 (55.5) 4 (3.7) 0 (0) 0 (0)
Fracture side, n (%) Right Left	58 (53.7) 50 (46.2)
AO/OTA classification, n (%) 31-A1 31-A2 31-A3	21 (19.4) 61 (56.4) 26 (24)
Nail length, n (%) 170 200 240 300 320 340 350 360 380	1 (0.9) 4 (3.7) 57 (52.7) 2 (1.8) 10 (9.2) 22 (20.3) 1 (0.9) 2 (1.8) 9 (8.3)
Blood transfusion, n (%) No Yes	62 (57.4) 46 (42.5)
Time of operation (minutes), mean (SD)	156.5 (60.2)
Hospital stay (days), mean (SD)	7.5 (3.9)
Time to mobilization (days), mean (SD)	164.8 (174.3)
Time to union (months), mean (SD)	7.4 (12.1)
Lateral wall fracture, n (%) No Yes	71 (65.7) 37 (34.2)
Fracture gap (mm), mean (SD)	7.7 (3.5)
Lateral wall fragment, n (%) No Yes	88 (81.4) 20 (18.5)
TAD (mm), median (SD)	17.7 (5.4)
TAD, n (%) <25 mm ≧25 mm	96 (88.8) 12 (11.1)
Parker’s ratio in AP%, mean (SD)	38.2 (8.1)
Parker’s ratio in lateral%, mean (SD)	38.9 (8.2)
Distance between posterior tip of blade to lateral cortex (mm), mean (SD)	6.6 (4.2)
The new postoperative stability score, mean (SD)	6.5 (1.1)

**Table 2:** Comparison of patient characteristics and technical variables in patients with loss of reduction.

Variable	No Loss of Reduction Group (N = 85)	Loss of Reduction Group (N = 23)	*p* Value
Age years, mean (SD)	81.7	83.7	0.838
Male gender (%)	25 (29.4)	7 (30.4)	0.924
BMI	22.8	24.3	0.278
ASA classification (%)			0.780
I	1 (1.1)	0 (0)	
II	32 (37.6)	11 (47.8)	
III	49 (57.6)	11 (47.8)	
IV	3 (3.5)	1 (4.3)	
V	0 (0)	0 (0)	
VI	0 (0)	0 (0)	
AO/OTA classification (%)			0.407
31-A1	14 (16.4)	7 (30.4)	
31-A2	51 (60)	10 (43.4)	
31-A3	20 (23.5)	6 (26)	
Fracture side (%)			0.268
Right	48 (56.4)	10 (43.4)	
Left	37 (43.5)	13 (56.5)	
Nail length (%)			0.461
170	1	0	
200	4	0	
240	47	10	
300	2	0	
320	6	4	
340	15	7	
350	1	0	
360	1	1	
380	8	1	
Blood transfusion (%)			0.071
No	45 (52.9)	17 (73.9)	
Yes	40 (47)	6 (26)	
Time of operation minutes, mean (SD)	155	162.2	0.442
Hospital stay days, mean (SD)	7.6	7.2	0.122
Time to mobilization days, mean (SD)	165	164.3	0.716
Time to union months, mean (SD)	7.12	8.47	0.636
Lateral wall fracture (%)			0.952
No	56 (65.8)	15 (65.2)	
Yes	29 (34.1)	8 (34.7)	
Fracture gap mm, mean (SD)	7.62	8.27	0.125
Lateral wall fragment (%)			0.875
No	69 (81.1)	19 (82.6)	
Yes	16 (18.8)	4 (17.3)	
TAD, median (SD)	17.4	19	0.780
TAD (%)			0.374
<25 mm	76 (89.4)	19 (82.6)	
≧25 mm	9 (10.5)	4 (17.3)	
Parker’s ratio in AP%, mean (SD)	37.63	49.51	0.709
Parker’s ratio in lateral%, mean (SD)	38.42	40.85	0.814
Distance between posterior tip of blade to lateral cortex mm, mean (SD)	6.47	7.12	0.103
A new postoperative stability score, mean (SD)	6.68	4.83	0.045 *

* *p* value less than 0.05 was considered statistically significant.

**Table 3:** Multivariate analysis of patient demographics and radiologic parameters.

Varible	Odds Ratio (95% Confidence Interval)	*p* Value
Parker’s ratio in AP%	1.11 (0.996 to 1.243)	0.058
Parker’s ratio in lateral%	1.005 (0.929 to 1.087)	0.903
Tip apex distance < 25 mm	0.675 (0.080 to 5.677)	0.717
The new postoperative stability score	0.076 (0.022 to 0.263)	*p* < 0.05

## Data Availability

Data is unavailable due to ethical restrictions.
